# Prevalence of Metabolic Syndrome and Associated Risk Factors in the United Arab Emirates: A Cross-Sectional Population-Based Study

**DOI:** 10.3389/fpubh.2021.811006

**Published:** 2022-01-24

**Authors:** Ibrahim Mahmoud, Nabil Sulaiman

**Affiliations:** ^1^Department of Family and Community Medicine and Behavioral Sciences, University of Sharjah College of Medicine, Sharjah, United Arab Emirates; ^2^Clinical Diabetes and Epidemiology Research Group, Baker Heart and Diabetes Institute, Melbourne, VIC, Australia

**Keywords:** blood glucose, blood pressure, metabolic syndrome, MetS, obesity

## Abstract

**Aims:**

To estimate the prevalence of metabolic syndrome (MetS) and its associated risk factors among the United Arab Emirates (UAE) residents.

**Methods:**

A cross-sectional population-based study was conducted among adults living in Sharjah and Northern Emirates using the UAE National Diabetes and Lifestyle Study (UAEDIAB) data. Anthropometric measurements and fasting blood samples were obtained. The National Cholesterol Education Program's Adult Treatment Panel III (NCEP/ATP III) guidelines were used to define metabolic syndrome (MetS).

**Results:**

A total of 3,212 subjects (74.1% men, 25.9% women, mean age 39 ± 11.3 years old) were included in this study. The overall prevalence of MetS was 37.4% (32.7% in women and 39% in men). The prevalence was 33.6% in the Emirati population (38.7% in women and 28.8% in men), 34.5% in the Arab non-Emirati population (29.8% in women and 36.3% in men) and 40.7% in the Asian non-Arab population (25.8% in women and 43.1% in men). Age, gender, ethnicity, educational level, marital status and body mass index were positively associated with MetS.

**Conclusions:**

This study indicates a high burden of MetS in the UAE, particularly among Emirati women and Asian non-Arab men. The young adult population in the UAE has a high prevalence of MetS compared to global estimates in the same age group. Aggressive intervention strategies targeting the whole population as well as individuals at a high risk are recommended to prevent the development of cardiovascular diseases.

## Introduction

Metabolic syndrome (MetS) is a multifactorial condition that is characterized by a combination of three or more of the following risk factors: increased waist circumference, high triglycerides, low high-density lipoprotein-cholesterol (HDL-C), high blood pressure and high fasting blood glucose (1, 2). The prevalence of MetS is increasing and is estimated to be over 20% among the adult population worldwide, causing huge health, social and economic burdens (2, 3).

Epidemiological studies have shown that MetS is strongly associated with accelerated atherosclerosis, which considerably increases the rates of atherosclerotic events, including myocardial infarction and stroke, and increases the risk of cardiovascular mortality (4–6). In the United Arab Emirates (UAE), myocardial infarction and strokes represent the first- and third-most common causes of premature death, respectively, and combined, they caused ~40% of all deaths in 2017 (7, 8). Recently, increasing rates of all five components of MetS have been reported in the UAE. The prevalence of obesity in adults in the UAE steadily increased from ~22% in 2007 to 30% in 2016 (9) and is expected to reach up to 37% by 2025 (10). A recent study showed that up to 40% of the UAE population was affected by either prediabetes or diabetes (11). Furthermore, the rates of overall dyslipidemia (73%) (12) and hypertension (31%) (13) in the UAE were also reported to be higher than the global rates (14). Overall, a large sample study from all UAE residents reported a high prevalence of MetS (40%) (15). However, the rates might differ across the emirates due to differences in the nature of the populations and risk factors encountered.

The National Cholesterol Education Program Adult Treatment Panel III (NCEP ATP III) guidelines (16), which contain MetS criteria, are commonly used worldwide to estimate the prevalence of MetS. The aim of this study was to estimate the prevalence of MetS among Emirati and non-Emirati adult populations using NCEP ATP III criteria. An additional aim was to describe the risk factors associated with MetS in the study population.

## Methods

### Study Design, Participants, and Setting

This study used secondary data from the UAE National Diabetes and Lifestyle Study (UAEDIAB). The UAEDIAB study was a cross-sectional population survey conducted to examine the prevalence of diabetes mellitus and its associated risk factors among Emirati and non-Emirati populations. Anthropometric measurements and fasting blood samples were also obtained. The UAEDIAB study recruited participants who had been living in Sharjah, Dubai and the Northern Emirates (Fujairah, Ajman, Ras al-Khaimah, and Umm al-Qaiwain) for at least four years. The study population was selected using a multistage systematic random sampling technique. People with serious physical disabilities, learning disorders and communication barriers, as well as pregnant women, were excluded from the study. The details of the survey methods and sampling of the UAEDIAB study have been published elsewhere (17).

### Data Collection Procedure

Sociodemographic (gender, nationality based on country of origin, date of birth, education level and marital status) and self-reported lifestyle behaviors (smoking and physical activity) were obtained through a face-to-face interview using a questionnaire. Subjects were interviewed by two trained research assistants who recorded participants' responses using a standardized interview technique and a defined set of responses. Emiratis were interviewed in their homes, whereas non-Emiratis were interviewed in the Preventive Medicine Department during their visit to renew their residence visa. Confidentiality and privacy of participants were assured. After completion of the interview, anthropometric measurements and systolic and diastolic blood pressures were obtained.

### Definitions of Metabolic Syndrome and Study Variables

According to the NCEP ATP III, MetS was considered to be present if three or more of the following diagnostic criteria were identified (ethnicity-specific waist circumference cutoffs were used) (16):

- Increased waist circumference, ≥102 cm in men and ≥88 cm in women (≥90 cm in men and ≥80 cm in women of Asian ethnicity)
- Raised serum triglycerides, ≥1.7 mmol/l
- Reduced serum HDL-C, <1 mmol/l in men and <1.3 mmol/l in women
- Raised fasting plasma glucose, ≥5.6 mmol/l
- Raised blood pressure, systolic ≥130 mmHg or diastolic ≥ 85 mmHg

WC was measured, based on the WHO STEPwise Approach to Surveillance (STEPS) protocol (18), at the midpoint between the lower margin of the lowest palpable rib and the top of the iliac crest using a non-stretchable plastic tape. An automated sphygmomanometer was used to take blood pressure measures; the individual was seated in a chair for around 3 min before being measured. Fasting blood samples were assayed to determine the plasma glucose (FBG), serum triglycerides and serum HDL-C.

### Bias

To standardize the data collection procedure, all data collectors attended a comprehensive training workshop that included interview techniques, data collection tools, practical applications and field guidelines. For WC, each measurement was repeated twice; if the measurements were within 1 cm of one another, the average was calculated, if the difference between the two measurements exceeded 1 cm, the two measurements were repeated (18). Blood pressure was measured three times at 10-min intervals. The average of all three measurements was considered the most accurate and was recorded.

### Ethics Approval

Both the University of Sharjah ethics committee and the Ministry of Health and Prevention Research Ethics Committee approved this study (MOHP/DXB/RE-SUBC/NO-12/2016). Each subject read the study information sheet and signed an informed consent form prior to participation.

### Statistical Analysis

Current study accessed a secondary data of 3,212 subjects with complete MetS information from the UAEDIAB Study. In this study, bivariate analyses were conducted using Pearson's chi-squared test to identify significant differences in prevalence of the MetS components according to gender and factors associated with MetS. Multivariate binary logistic regression analysis using the enter method was conducted for the significant predictors of MetS, with the primary outcome being MetS status (yes/no). All of the variables that were statistically significant in bivariate analyses were included in the regression model. The Statistical Package for Social Sciences (SPSS) version 26 (IBM Corp, New York) was used to perform analyses.

### Patient and Public Involvement

Patients or the public were not involved in the design, conduct, reporting or dissemination the findings of this study.

## Results

Of the 3,530 patients who were eligible to participate in the study, 3,212 (91%) had complete MetS data, with 2,380 (74.1%) being men and 832 (25.9%) being women. The mean age of the entire study population was 39 years (SD ± 11.3 years).

Table 1 shows the prevalence of MetS and its five components among the study participants according to gender. Women had a significantly higher prevalence of increased waist circumference and lower HDL-C than men, 67 vs. 53.7% and 44.1 vs. 36.3%, respectively, while men had significantly higher rates of high blood pressure and increased triglycerides than women, 53 vs. 33% and 39.5 vs. 21.9%, respectively. The overall prevalence of MetS in this study was 37.4%, with a significantly higher rate among men (39%) than women (32.7%; *p*-value = 0.001; Table 1).

**Table 1:** Overall prevalence of the MetS and its five components in the study population stratified by gender, *n* (%, 95% confidence interval).

**MetS component**	**Gender**		***P*-value**	**Overall**
	**Female**	**Male**		
Increased WC	564 (67.0, 63.7–70.1)	1,282 (53.7, 51.7–55.7)	**<0.001**	1,846 (57.2, 55.4–58.8)
Raised serum TG	199 (21.9, 19.3–24.7)	1,035 (39.5, 37.7–41.4)	**<0.001**	1,234 (35.0, 33.4–36.6)
Reduced serum HDL-C	399 (44.1, 40.9–47.3)	951 (36.3, 34.5–38.2)	**<0.001**	1,350 (38.3, 36.7–39.9)
Raised BP	297 (33.0, 30.0–36.1)	1,386 (53.0, 51.1–55.0)	**<0.001**	1,683 (48.0, 46.3–49.6)
Raised BG	283 (31.1, 28.2–34.2)	773 (29.5, 27.8–31.3)	0.366	1,056 (30.0, 28.5–31.5)
MetS	272 (32.7, 29.6–36.0)	929 (39.0, 37.1–41.0)	**0.001**	1,201 (37.4, 35.7–39.1)

*P-values in bold are statistically significant. BG, Blood glucose; BP, Blood pressure; HDL, High-density lipoprotein; TG, Triglycerides; WC, waist circumference; MetS, metabolic syndrome*.

**Figure 1:**
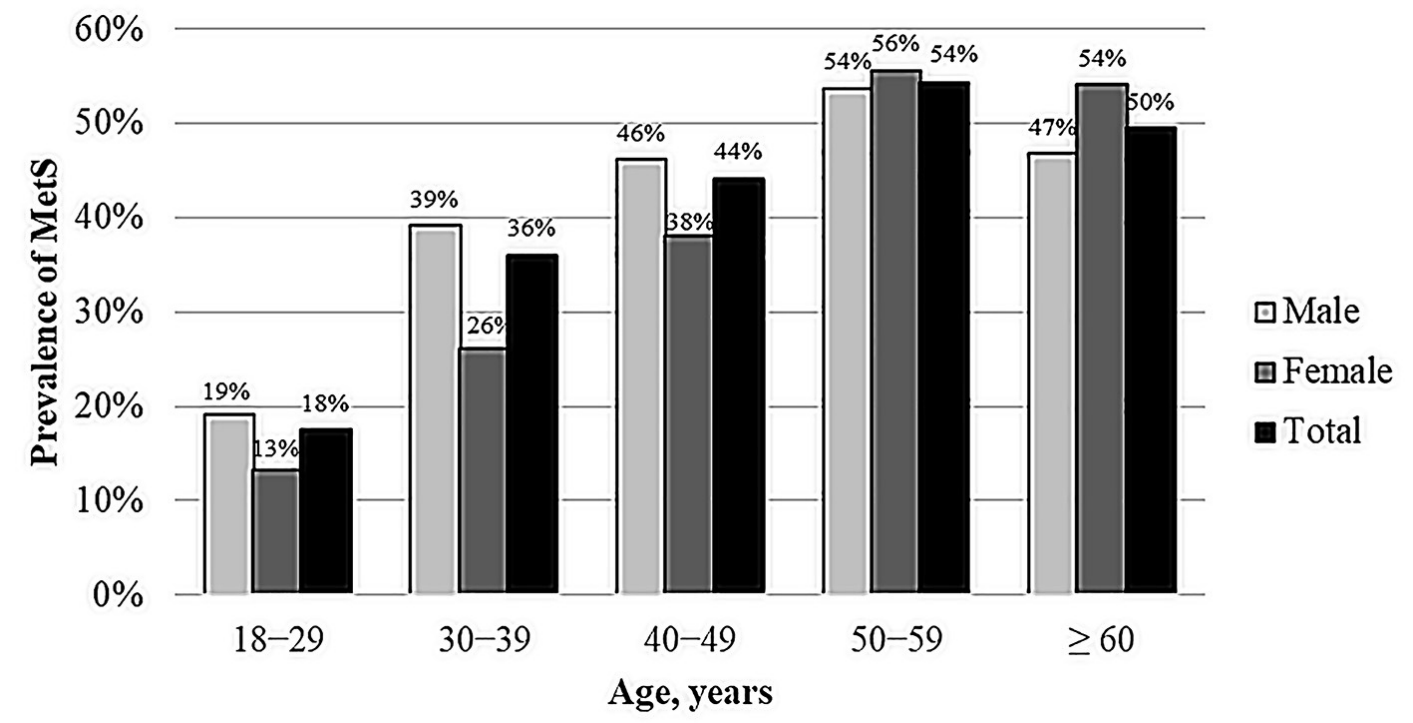
Gender and age-specific prevalence of metabolic syndrome.

Figure 1 shows the gender- and age-specific rates of MetS. Overall, the prevalence of MetS is higher in men under 50 years old than women under 50 years old, while the prevalence is higher in women over 50 years old than men over 50 years old.

Table 2 shows the MetS status (no/yes) based on the study population's sociodemographic characteristics. Bivariate analyses in Table 2 show that gender, age, ethnicity, marital status, educational level and body mass index were significantly associated with MetS (*p* ≤ 0.05). Smoking and physical activities were not significantly associated with MetS in the bivariate analyses of the entire study sample (*p* > 0.05; Table 2).

**Table 2:** Prevalence of the MetS based on the characteristics of the study population, *n* (%).

**Variable**	**Metabolic** **syndrome status**		***P*-value**	**Total**
	**No**	**Yes**		
**Gender**				
Female	560 (67.3)	272 (32.7)	**0.001**	832 (25.9)
Male	1,451 (61)	929 (39)		2,380 (74.1)
**Age, years**				
18–29	517 (82.5)	110 (17.5)	**<0.001**	627 (19.5)
30–39	776 (64)	437 (36)		1,213 (37.8)
40–49	444 (55.9)	350 (44.1)		794 (24.7)
50–59	174 (45.8)	206 (54.2)		380 (11.8)
≥ 60	100 (50.5)	98 (49.5)		198 (6.2)
**Ethnicity**				
Emirati	532 (66.4)	269 (33.6)	**<0.001**	801 (24.9)
Arab non-Emirati	406 (65.5)	214 (34.5)		620 (19.3)
Asian non-Arab	1,014 (59.3)	695 (40.7)		1,709 (53.2)
Others	59 (72)	23 (28)		82 (2.6)
**Marital status**				
Single	472 (81.4)	108 (18.6)	**<0.001**	580 (18.1)
Married	1,477 (73.6)	1,033 (41.2)		2,510 (78.3)
Divorced/widowed /separated	59 (50.4)	58 (49.6)		117 (3.6)
**Education level**				
Primary	284 (53.8)	244 (46.2)	**<0.001**	528 (16.4)
Secondary	743 (61.3)	469 (38.7)		1,212 (37.7)
Tertiary	984 (66.8)	488 (33.2)		1,472 (45.9)
**Body mass index**				
Normal	613 (83.4)	122 (16.6)	**<0.001**	735 (22.9)
Overweight	875 (63.2)	509 (36.8)		1,384 (43.1)
Obese	521 (47.8)	568 (52.2)		1,089 (34)
**Physical activity status**				
No	1,695 (62.3)	1,027 (37.7)	0.326	2,722 (85.1)
Yes	307 (64.6)	168 (35.4)		475 (14.9)
**Smoking status**				
No	1,631 (63)	958 (37)	0.257	2,589 (81.6)
Yes	352 (60.5)	230 (39.5)		582 (18.4)

*P-values in bold are statistically significant*.

**Figure 2:**
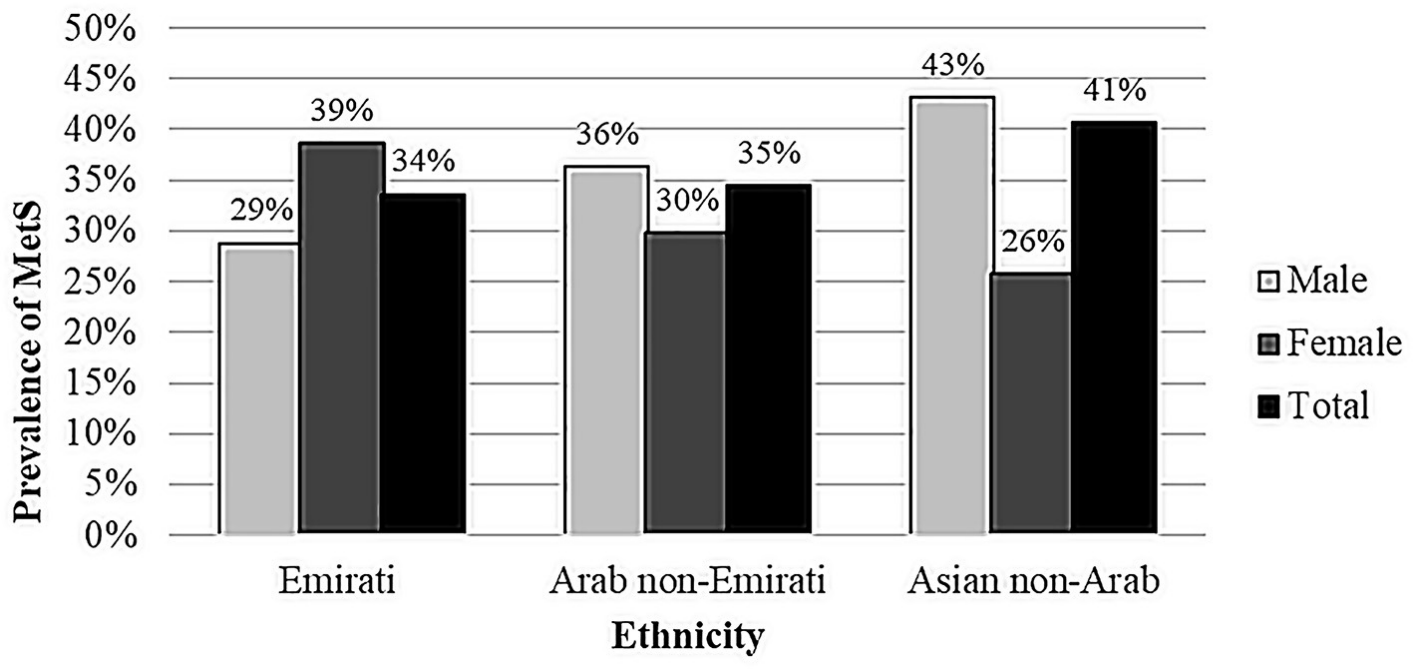
Gender and ethnicity-specific prevalence of metabolic syndrome.

Figure 2 illustrates the prevalence of MetS (rounded to nearest one) by gender for different ethnicities. The prevalence was 33.6% in the Emirati population (38.7% in women and 28.8% in men, 0.003), 34.5% in the Arab non-Emirati population (29.8% in women and 36.3% in men, *p* = 0.129) and 40.7% in the Asian non-Arab population (25.8% in women and 43.1% in men, *p* < 0.001).

Table 3 shows significant factors associated with MetS among the entire study population. Older age [odds ratio (OR), 95% confidence interval (CI) = 2.02 (1.54–2.66); 2.64, (1.96–3.55, *p* < 0.0001); 3.95 (2.82–5.54, *p* < 0.0001); 3.27 (2.16–4.95, *p* < 0.0001)], being from an Asian non-Arab population [1.60 (1.29–1.98, *p* < 0.0001)], being married or divorced/widowed/separated [1.47 (1.12–1.93, 0.006); 2.26 (1.35–3.80, *p* = 0.002)] and being overweight or obese [2.93 (2.31–3.71, *p* < 0.0001); 5.39 (4.23–6.87, *p* < 0.0001)] were significantly associated with a high risk of MetS compared to young age, Emirati ethnicity, single marital status and normal weight, respectively. Tertiary education attainment was a protective factor against MetS (OR 0.66, 95% CI 0.52–0.84, *p* = 0.001) compared to primary education attainment (Table 3).

**Table 3:** Binary logistic regression model for the predictors of metabolic syndrome in whole UAE residents, OR (95% CI).

**Variable**	**OR (95% CI)**	***P*-value**
**Gender**		
Male	Reference	
Female	0.84 (0.68–1.03)	0.087
**Age, years**		
18–29	Reference	
30–39	2.02 (1.54–2.66)	**<0.001**
40–49	2.64 (1.96–3.55)	**<0.001**
50–59	3.95 (2.82–5.54)	**<0.001**
≥ 60	3.27 (2.16–4.95)	**<0.001**
**Nationality**		
Emirati	Reference	
Arab non-Emirati	1.15 (0.89–1.49)	0.287
Asian non-Arab	1.60 (1.29–1.98)	**<0.001**
Others	1.18 (0.68–2.04)	0.557
**Marital status**		
Single	Reference	
Married	1.47 (1.12–1.93)	**0.006**
Divorced/Widowed/separated	2.26 (1.35–3.80)	**0.002**
**Education level**		
Primary	Reference	
Secondary	0.86 (0.68–1.09)	0.204
Tertiary	0.66 (0.52–0.84)	**0.001**
**Body mass index**		
Normal	Reference	
Overweight	2.93 (2.31–3.71)	**<0.001**
Obese	5.39 (4.23–6.87)	**<0.001**
**Physical activity status**		
No	Reference	
Yes	0.95 (0.76–1.19)	0.659
**Smoking status**		
No	Reference	
Yes	1.23 (1.00–1.52)	0.052

*P-values in bold are statistically significant. OR, odds ratio; CI, confidence interval*.

Table 4 demonstrated that the factors associated with MetS were different among different ethnicities. Among the Emirati population, women had a higher risk of MetS than men (OR 1.68, 95% CI 1.17–2.42, *p* = 0.005], while among the Asian non-Arab population, men had a higher risk of MetS than women (OR 0.43, 95% CI 0.31–0.60, *p* < 0.0001), and no gender difference was observed among the Arab non-Emirati population. Furthermore, tertiary education attainment was only a protective factor against MetS in the Emirati and Asian non-Arab populations. Smoking was significantly associated with a high risk of MetS only in the Arab non-Emirati population [OR 1.69, 95% CI 1.11–2.57, *p* = 0.015; Table 4).

**Table 4:** Binary logistic regression models for the predictors of metabolic syndrome by ethnicity, OR (95% CI).

**Variable**	**Emirati**		**Arab non-emirati**		**Asian non-Arab**	
	**OR (95% CI)**	***P*-value**	**OR (95% CI)**	***P*-value**	**OR (95% CI)**	***P*-value**
**Gender**						
Male	Reference		Reference		Reference	
Female	1.68 (1.17–2.42)	**0.005**	0.71 (0.45–1.14)	0.158	0.43 (0.31–0.60)	**<0.001**
**Age, years**						
18–29	Reference		Reference		Reference	
30–39	1.36 (0.73–2.54)	0.332	2.06 (1.04–4.23)	**0.049**	2.56 (1.57–3.21)	**<0.001**
40–49	1.73 (0.91–3.31)	0.095	3.32 (1.53–7.20)	**0.002**	2.70 (1.83–3.98)	**<0.001**
50–59	1.69 (0.83–3.45)	0.149	5.08 (2.19–11.83)	**<0.001**	5.27 (3.28–8.46)	**<0.001**
≥ 60	1.28 (0.56–2.89)	0.561	4.96 (1.86–13.81)	**0.001**	6.35 (3.03–13.30)	**<0.001**
**Marital status**						
Single	Reference		Reference		Reference	
Married	1.82 (0.98–3.37)	0.057	2.16 (1.06–4.39)	**0.034**	1.28 (0.89–1.84)	0.189
Divorced/widowe/separated	2.36 (1.10–5.19)	**0.032**	3.33 (0.76–14.53)	0.109	2.90 (0.78–10.80)	0.112
**Education level**						
Primary	Reference		Reference		Reference	
Secondary	0.75 (0.44–1.27)	0.289	1.04 (0.43–2.54)	0.938	0.79 (0.59–1.05)	0.113
Tertiary	0.56 (0.32–0.94)	**0.038**	0.79 (0.34–1.83)	0.582	0.63 (0.46–0.85)	**0.003**
**Body mass index**						
Normal	Reference		Reference		Reference	
Overweight	1.74 (1.13–2.66)	**0.011**	4.26 (1.91–9.43)	**<0.001**	4.21 (3.02–5.89)	**<0.001**
Obese	1.91 (1.25–2.91)	**0.003**	15.60 (7.04–34.55)	**<0.001**	7.83 (5.50–11.15)	**<0.001**
**Physical activity status**						
No	Reference		Reference		Reference	
Yes	0.74 (0.43–1.28)	0.281	0.86 (0.52–1.43)	0.552	1.05 (0.78–1.42)	0.738
**Smoking status**						
No	Reference		Reference		Reference	
Yes	1.26 (0.77–2.05)	0.355	1.69 (1.11–2.57)	**0.015**	1.13 (0.83–1.53)	0.473

*P-values in bold are statistically significant. OR, odds ratio; CI, confidence interval*.

## Discussion

In the current study, the overall prevalence of MetS based on NCEP ATP III criteria was 37% (34% for the Emirati population, 35% for the Arab non-Emirati population and 41% for the Asian non-Arab population). According to the NCEP ATP III criteria, the prevalence of MetS in this study was slightly lower than the national reported rate of 40% (15) and much lower than the reported rate in Abu Dhabi emirate, the capital of the UAE (50%) (19). However, the prevalence in the current study was higher than most of the reported rates in the other Gulf Cooperation Council countries, such as Kuwait (22%), Oman (25%), Saudi (28%) and Qatar (29%) (20).

In this study, there were some variations in MetS prevalence in different age groups, genders and ethnic groups. Aging is well-recognized as a major risk for MetS and its components. In the current study, the prevalence of MetS increases significantly with age: the prevalence doubled from 18% in young adults (18–29 years) to 36% in the second decade of adulthood (30–39 years) and reached over 50% after the age of 50 years. Alarmingly, the prevalence of MetS among the young population in this study (18%) was triple the global prevalence estimate, which ranges from 5 to 7% using NCEP ATP III criteria (21).

Overall, the prevalence of MetS in this study was higher among men than among women. Men had a slightly higher prevalence of MetS than women before the age of 50 years, while women had a higher prevalence than men after 50 years of age. Furthermore, this study revealed a higher prevalence of MetS in the Asian non-Arab population (41%) compared to Emirati, Arab non-Emirati and other ethnicities, which is consistent with previous studies that demonstrated that Asian populations have a high burden of MetS (22, 23). MetS prevalence was higher in Emirati women than in Emirati men, while it was higher in Asian non-Arab men than in Asian non-Arab women, and no significant gender difference was observed in Arab non-Emirati. These differences in MetS prevalence might be due to variations in genetics and risk factors faced by each group. For instance, in the Northern Manhattan Family Study among Caribbean-Hispanic families, the heritability of MetS was determined to be 24% (24). Furthermore, familial partial lipodystrophy is a monogenic disease caused by mutations in several genes linked to hyperglycemia, dyslipidemia, and hypertension (25). In the UAE, a couple of studies found links between MetS and particular genetic and metabolic risk factors among the country residents (26, 27). According to a systematic review of a number of epidemiological studies, vitamin D levels have been linked to the progression and prognosis of a variety chronic diseases including MetS components (28). Individual vitamin D status may be influenced by ethnic genetic variability, which may affect vitamin D synthesis, transportation, and metabolism (29). Al Safara et al. reported that the vitamin D receptor gene is linked to hyperglycemia susceptibility in Emiratis (30).

Our study also identified differences in the prevalence of MetS components in men and women. In this study, increased waist circumference and reduced serum HDL-C were highly prevalent among women, while raised serum triglyceride and raised blood pressure were highly prevalent among men, and difference in prevalence of raised blood glucose between men and women. This is consistent with studies that showed that Emirati women are at increased risk of central obesity and reduced HDL-C blood levels (31) and that Asian non-Arab men are at increased risk of high blood pressure (13). The Emirati community has undergone a fast socioeconomic growth in recent decades, which led to a sedentary lifestyle, including a lack of physical activity and unhealthy dietary habits, which are highly associated with obesity and dyslipidemia (32). Due to the conservative nature of the UAE population and the lack of culturally sensitive exercise facilities in the past, Emirati women were less prone to participate in physical activities (33). However, this might not be a barrier anymore as there are now several gender-specific facilities throughout the country, as well as the availability of home-friendly physical activity equipment (34). On other hand, the majority of Asian male workers from India, Pakistan and Bangladesh are unskilled workers with low education levels and working in stressful conditions, which puts them at a higher risk for developing metabolic risk factors such as high blood pressure (13). This is consistent with the study finding that low education attainment was associated with increased risk of MetS.

Overall, smoking and alcohol consumption were not significantly associated with MetS in this study. These factors might be under-reported in a conservative community like that of the UAE, particularly among women. However, smoking was significantly associated with MetS among the Arab non-Emirati population.

From a public health perspective, our study suggests that the UAE populations are at great risk of developing MetS and thereby increasing the burden of cardiovascular diseases in the UAE. Therefore, regular screening tests for those groups may be useful in detecting MetS components at an early stage before development of MetS with three components. Moreover, early intervention strategies to prevent and treat MetS in these high-risk groups could have significant public health benefits in decreasing the burden of cardiovascular disease in the UAE.

The strength of this study was its reporting of an up-to-date prevalence of MetS in the UAE using fasting blood samples in a large representative sample. However, this study did have some limitations. This study might not be generalizable to all UAE residents, as it was limited to the Northern Emirates population. In addition, causality could not be established in this study due to the cross-sectional design. Moreover, our study relied on self-reporting for physical activity level, which may have introduced bias. Furthermore, this study did not assess some important risk factors of MetS that have been identified in the literature, such as alcohol consumption and unhealthy diet.

## Conclusion

The prevalence and risk factors of MetS vary across age groups, gender and ethnic groups in the UAE. This study indicates a high burden of MetS in the UAE, particularly among Emirati women and Asian non-Arab men. The young adult population has a much higher prevalence of MetS than global estimates in the same age group. Therefore, aggressive intervention strategies targeting the whole population as well as individuals at a higher risk are recommended to prevent the development of cardiovascular diseases.

## Data Availability Statement

The raw data supporting the findings of this study are available from the corresponding author on request.

## Ethics Statement

The studies involving human participants were reviewed and approved by both the University of Sharjah Ethics Committee and the Ministry of Health and Prevention Research Ethics Committee approved this study (MOHP/DXB/RE-SUBC/NO-12/2016). Each subject read the study information sheet and signed an informed consent form prior to participation. The patients/participants provided their written informed consent to participate in this study.

## Author Contributions

IM conducted statistical analysis, interpretation of data, and drafted the manuscript. NS conceived and designed the study. Both authors critically revised the manuscript and gave final approval of the present version to be submitted.

## Funding

This work was supported by Ministry of Health and Prevention, University of Sharjah and Sanofi (grant number 120301).

## Conflict of Interest

The authors declare that the research was conducted in the absence of any commercial or financial relationships that could be construed as a potential conflict of interest.

## Publisher's Note

All claims expressed in this article are solely those of the authors and do not necessarily represent those of their affiliated organizations, or those of the publisher, the editors and the reviewers. Any product that may be evaluated in this article, or claim that may be made by its manufacturer, is not guaranteed or endorsed by the publisher.
